# 

**Published:** 1899-10-28

**Authors:** 


					THE HOSPITAL. ?? The standard of highest purity."? .ancet. Octmbrr 28th, 1899.
CADBURY'S H ** CADBURY'S
COOOA ^ COCOA
ABSOLUTELY PURE. ~ ~v/ NO DRUGS OR ALKALIES USED.
0
? w lazuutt nt&i trt/v inrmMMnr
No. 683. Vol. XXVII. Satubday,
Oct. 28tii, 1899.
[SubscriptionJermB.j FR1QJ. TWOPENCE.

				

